# Evaluation of Nurses' Perceptions on Providing Patient Decision Support with Cardiopulmonary Resuscitation 

**DOI:** 10.5402/2012/591541

**Published:** 2012-12-05

**Authors:** Nicole Pyl, Prudy Menard

**Affiliations:** The Ottawa Hospital, General Campus, 501 Smyth Road, Ottawa, ON, Canada K1H 8L6

## Abstract

The decision whether to receive cardiopulmonary resuscitation (CPR) is a decision in which the personal values of the patient must be considered along with information about the risks and benefits of the treatment. A decision aid can be used to provide patient decision support to a patient who is seriously ill and needs to consider CPR options. The goal of this project was to identify the barriers and facilitators to using a CPR decision aid, through evaluating nursing perceptions on providing patient decision support. Using a needs assessment, it was determined that implementing a patient decision aid for CPR status in the Acute Monitor Area (AMA) of The Ottawa Hospital would be an excellent quality improvement project. The nurses who chose to participate were given an education session regarding patient decision support. Questionnaires were distributed to evaluate their views of patient decision support and decision aids before and after the education session and implementation of the CPR decision aid. Questionnaire results did not indicate a significant change between before or after education session and decision aid implementation. Qualitative reports did indicate that nurses generally have positive attitudes toward patient decision support and decision aids. The nurses identified specific barriers and facilitators in their commentaries. This clinically relevant data supports the idea that patient decision support should be integrated into daily nursing practice.

## 1. Introduction

Improving informed decision making is essential for supportive end of life care [1–5]. Cardiopulmonary resuscitation (CPR) preferences are the most common end of life discussion but occur infrequently and vary in content [1, 2]. Patients may not have the basic information needed and the timing for the discussion may be inappropriate [6–8]. Patient decision support focuses on providing the patient and families with practical information and resources.

CPR preferences may also be overlooked or set aside by practitioners because it is a value-sensitive decision or because it is not identified as a high priority discussion [1, 3, 6, 9]. Value-sensitive decisions would benefit from a patient decision aid, where the patient is recognized as an expert in judging his/her own values [10–13]. Patients who need to address CPR status would benefit from health care professionals comfortable and familiar with providing patient decision support.

CPR status is one of the most important health decisions and requires careful consideration of all alternatives and the consequences. For seriously ill patients, CPR preferences are commonly set aside and communication between the patient, family, and health care team is lacking information and followup [2]. Nursing influencing factors using patient decision support for CPR status needs to be evaluated.

## 2. Methods

### 2.1. Setting

This project took place at The Ottawa Hospital, Acute Monitor Area (AMA) Unit. This six-bed unit specializes in acute care, managing patients with a variety of complex medical conditions such as chronic obstructive pulmonary disease, congestive heart failure, pneumonia, and multisystem failure disorders. 

### 2.2. Goal

The goal of this project was to implement a publically available patient decision aid for CPR status and to identify any factors which limit or encourage its use [14]. The objectives were to identify nursing perceptions regarding patient decision support before and after using a patient decision aid regarding CPR, clarify any barriers and facilitators influencing the provision of a patient decision aid regarding CPR, and determine whether nursing perceptions of patient decision support can be positively influenced with a brief, theory-based, skill building educational intervention. This pilot project has focused on the efficacy of providing nursing specific patient decision support.

## 3. Literature Review

### 3.1. Search Strategy

A literature review was conducted to identify scholarly English publications pertaining to “cardiopulmonary resuscitation preferences,” “end of life treatment,” “patient decision support,” “decision making,” and “patient decision aids” in PubMed, PsycINFO, CINAHL, Proquest Nursing and Allied Health, and the Cochrane Database of Systematic Reviews. Notable articles were screened by reviewing their reference lists for relevant publications. Grey literature searches were also conducted through the Registered Nurses' Association of Ontario (RNAO), College of Nurses of Ontario (CNO), and the Ottawa Hospital Research Institute (OHRI) websites.

### 3.2. CANHELP

It has been identified by Heyland and colleagues [3] through the Canadian Health Care Evaluation Project (CANHELP) that better planning for end of life care including enhanced relationships with physicians and improving communication and decision making needs to be addressed in many Canadian hospitals. Reliable information regarding the patient's condition, understandable explanations for their situation, and addressing patients psychosocial feelings were also rated as high priorities [3]. The CNO practice guideline, Guiding Decisions about End-of-Life Care [1] indicates that clear communication between the patient, nurse, and the interprofessional team facilitates implementation of patient's wishes regarding end of life treatment. It is evidenced that a comprehensive and consistent way to address CPR status for seriously ill patients should be established and facilitated in the Canadian hospitals.

### 3.3. CPR Status

It has been determined that seriously ill patients have poor knowledge about what CPR entails and their role in the decision making process regarding their CPR status [6, 15]. Participants in any decision may not have all the necessary information to make an informed choice and the timing of the discussion may be inappropriate [6, 8]. CPR in particular is a sensitive subject in which all involved in the discussion can appraise differently, even the health care team [9]. Value sensitive issues are better addressed using a complete and understandable approach. 

### 3.4. Decision Making

The literature on patient decision support has increased a great deal since O'Connor and colleagues published their work in 1998 [10]. She has become a leader in the patient decision support realm. Informed decision making in health care is important. Patients want to be educated about their treatments and have an autonomous role in their care [5, 16]. There is a stress on the importance of knowing options and being able to provide that information to patients [16, 17]. Nurses have a unique relationship with patients; they can provide valuable support when they are faced with difficult decisions.

### 3.5. Decisional Conflict

Decisional conflict means that there is uncertainty about which course of action to take [10, 18]. Many issues contribute to decisional conflict and patients are likely to experience uncertainty to some degree when a decision is difficult to weigh [19]. When one needs to consider risk, loss, or a challenge to their personal values conflict can arise. Improving informed decision making is essential for supportive end of life care where the patient's goals of care are clear and communicated, thus reducing uncertainty [1, 3–5, 20]. 

### 3.6. Patient Decision Aids

Patient decision aids are a part of providing decision support. They are ‘‘tools that help people become involved in decision making” [18]. Patient decision aids reduce uncertainty, improve knowledge, generate realistic expectations, and clarify personal values [21]. These are evidence-based tools that can include outcome statistics and patient experiences for a variety of disease specific issues. They help people in making difficult decisions that are consistent with their personal values [12, 22]. 

### 3.7. Nurse Involvement

Facilitation of nurse involvement in end of life care is essential for comprehensive care. However, shared decision making has not been embraced by all health professionals and barriers have been identified which limit the use of patient decision aids [23]. An evaluation of nurses' perceptions with the use of a CPR patient decision aid can clarify issues with its use and can lead to sustainable utilization of the aid [13, 24, 25]. 

### 3.8. Conceptual Framework

The Ottawa Decision Support Framework ODSF (1998) developed by Annette O'Connor and colleagues guided this project. This concrete, midrange theory focuses on decisional needs, decision quality, and decision support. It can guide gaining decision support skills through a practical and structured approach [10, 18, 26]. It uses a three-step process to “assess client and practitioner determinants of decisions to identify decision support needs, provide decision support tailored to client needs, and evaluate the decision making process and outcomes” [27]. The theoretical underpinnings of the ODSF include decision theories in economics [28], psychology [29], social psychology [30], decisional conflict [31], and social support [32, 33]. 

The main assumption of the ODSF is that patients will likely select the choice that they believe is their best alternative which aligns with their personal values [10, 26]. Access to all the necessary resources to realize their choice through clear information when the issues are discussed is also a part of the main assumption [10, 26]. Personal (patient) and practitioner characteristics influence the decision making process as well, but knowledge is a key [10]. Providing information for patient options include specific information on benefits and harms, clarification of values associated with each option, ways to manage the views or pressures of others involved in the decision making process, and skills on implementing decisions [26]. Patient decision support includes providing information and coaching to improve knowledge and abilities. 

Patient decision support is provided through counselling, coaching, and decision aids [10]. Decision aids clarify values and address unmet decisional needs or conflict by asking individuals to identify personal importance of issues and evaluate each risk and benefit that influences their decision [18, 34]. Specifically, patient decision aids need to include the following elements: (1) information tailored to the patients health condition, (2) a value clarification exercise, (3) examples from other patients in similar circumstances, (4) guidance toward shared decision making, and (5) a medium such as a paper tool or interactive computer guide to present the information [12, 22]. They are not general guides for patients and they are not prescriptive in nature [35]. Patient decision aids can guide tailored decision support which focuses on patient's needs. 

Decision support is provided until decisional conflict is resolved and a quality decision is reached. Then aiding implementation and monitoring of the decision occur [4, 10]. During the evaluation stage of the ODSF, an understanding of the quality of decision making and outcomes is established [27, 34]. Informed decisions, ones that are consistent with personal values and that are determined by the patient to be the best alternative, are the result [10, 26]. In theory, the patient completing this process as intended will lead to overall satisfaction with their choice, quality of life, and adherence with their decision because it is informed by their values and resources [4, 34]. Quality decisions are informed by the best available evidence and are based on the values of the patient [22, 35].

Specifically, the ODSF guided this project to address unmet decisional needs or decisional conflict where uncertainty regarding the best choice for CPR status was identified in seriously ill patients. Realistic expectations were discussed, evidenced-based information was reviewed, support and resources were appraised, and patient values were considered [5, 10]. Appraising and articulating a patient's CPR wishes through clear communication and implementation are congruent with this framework [1]. The criteria involved in the ODSF are appropriate to the established project goal and objectives and thus are a straightforward justification for the choice of framework used. Through the provision of decision support using a CPR decision aid in combination with decision coaching and counselling, the framework was used to address decisional needs and uncertainty.

## 4. Intervention

### 4.1. Ethics Approval

Ethical approval for this project was obtained from The Ottawa Hospital Research Ethics Board. Consent was obtained from each participant and information regarding the project was provided. The intervention design involved three steps including (1) conducting a pretest, (2) educating the nurses on patient decision support and the CPR decision aid, and (3) conducting a posttest. 

### 4.2. Need Assessment

 It was identified during discussions with nurses who work in the AMA and their nurse educator (the project advisor) that frequently a patient's CPR status is not addressed in a timely manner. It was repeatedly suggested that an improvement needs to be made to address the patient's information and communication needs regarding CPR status. The discussions lead to an intervention focused on influencing nursing knowledge of patient decision support, uptake of a CPR decision aid, and identifying facilitators and barriers to its use [13, 23, 26, 36, 37]. Graham and colleagues [38] found that most health care practitioners are willing to use patient decision aids, given that adequate education and support are provided. 

### 4.3. Education Intervention

 Initially, an advertisement of the educational intervention was posted. All registered nurses who work in the AMA unit were approached to participate in the education session. Due to time constraints and that there were other quality improvement initiatives being implemented concurrently, it was decided that only a brief and basic education session would be offered. The education session consisted of an introduction to why the project was being implemented, what patient decision support is, and an overview of the CPR patient decision aid. Taking approximately 5–10 minutes, nurses were guided individually or in small groups through part one of the Ottawa Decision Support Tutorial, ODST [27], and the decision support aid [14]. Nurses were referred back to the ODST if more information was needed. 

### 4.4. After Education Session

After having received the education, the nurses were requested to provide patient decision support for CPR preference using the patient decision aid based on clinical opportunities and appropriateness [17, 26, 37]. Basic knowledge regarding patient decision support is needed to work effectively with patient decision aids [13, 23]. With background knowledge of patient decision support, the participants needed to identify using their clinical judgment if a patient would benefit from the CPR decision aid. Promotion of the patient decision aid was accomplished by going to the AMA unit frequently to check on uptake and being available to answer questions. Two posters were also strategically placed on the unit, acting as a reminder. 

### 4.5. Logistics

Each Ottawa Hospital form for code status was affixed with the CPR decision aid to prompt each nurse to its use. Nurses were advised that if a CPR decision aid was initiated and/or completed they were to write in the interprofessional progress notes in the patient's medical record of this. It was also asked that this information should be communicated to other team members in the patient's daily care plan.

## 5. Evaluation

### 5.1. Design

Both qualitative and quantitative measures were used to collect information. Before and after intervention questionnaires were the primary means of information collection. There were no pretested measurement tools found that fit the objectives of this project. Consequently, the questionnaires developed were influenced by a study conducted by Stacey and colleagues [13] to determine factors influencing decision support by call center nurses. For this project, the number of questions was reduced due to the project scope and objectives and was modified to fit the clinical setting. The questionnaire's design reflects the current literature on health care professionals' perceptions of patient decision support and decision aids [12, 23]. Qualitative observations and field notes were also routinely used for data collection [39]. 

### 5.2. Before and after Intervention Questionnaires

The questionnaires were designed to be clear and concise using a five-point Likert scale to encourage the participation in attaining data [40]. The questionnaires also have a section to generate nurses' views using an open-ended question format [40, 41]. Questionnaires were used because of their ability to gather data easily and for their capacity to examine notable differences in responses after the initiated intervention [42, 43]. The questionnaires were reviewed and revised with the project advisor prior to use. The postquestionnaire was conducted after six weeks of patient decision aid implementation.

### 5.3. Qualitative Evaluations

Qualitative observations and field notes were routinely collected in a designated journal. These observations were analyzed for recurring themes and notable results. Specifically responses were grouped into one of two categories, facilitators or barriers, and were grouped after each batch of questionnaires was received. Qualitative reports were used to gather data on the impacts on practice and participant's views on the project which may not be captured with the questionnaires [39]. Open-ended questions were directed to the AMA nurses as appropriate, such as “What do you think of patient decision support?” and “What has been your experience with patient decision support?” 

### 5.4. Procedures

Questionnaires were given to all AMA nurses who signed consent and agreed to participate in the project immediately before each education session. Then the education session was given. The after intervention questionnaires were given after six weeks of the first education session. All nurses had an average of five weeks or more to use the patient decision aid. All participants who agreed to complete the post questionnaire were entered into a draw for two gift baskets. Descriptive statistic methods were used to analyze questionnaire responses and content analysis was used in reviewing the qualitative reports [42]. 

## 6. Results and Discussion

### 6.1. Participant Statistics

There are currently 26 nurses who are trained to work in the AMA. Of those nurses, 3 were on maternity leave, 2 declined to participate and 21 agreed to take part in the educational session and before intervention questionnaire (*n* = 21). The age range of participants was 25–52 years and the average age of participants was 37.8 years. 16% of the participants were male. Most participants 81% worked in the AMA since inception (3 years). The average amount of nursing experience was 11.8 years. 

Not all nurses who initially agreed to take part in the project continued their participation. Sixteen agreed to participate in the after intervention questionnaire (*n* = 16/21). Participant characteristics were similar in comparison to the nonparticipant group. Age range was 27–52 years, average age was 37.8 years, 19% were male, and the average amount of nursing experience was 11.3 years. To participate in the after intervention questionnaire you must have received the education session and took the before intervention questionnaire. 

### 6.2. Questionnaire Results

Participants were asked to rate how strongly they agree or disagree with certain statements.  Table 1 identifies the results from the before intervention questionnaire. The results indicate that most respondents agree or strongly agree that using a patient decision aid would be beneficial to the patient and that more education should be directed toward nurses to provide decision support. The results were negatively skewed when asked if they felt confident in providing patient decision support.  Table 2 identifies the results from the after intervention questionnaire. Most nurses responded that they agree or strongly agree that decision aids for CPR was/is useful. 

### 6.3. Qualitative Results

The following points were recognized in the questionnaire and field note results for facilitators/benefits to patient decision support and aids:

- a team understanding of the patient condition and status, better communication,
- a standardized way to present information and a knowledge tool for nurses,
- supported by the literature, evidenced-based information,
- clear understanding of what CPR is and the risks/benefits,
- support for when patient is not able to make their own decision (family involved).

The following points were recognized in the questionnaire and field notes results for barriers/limitations to patient decision support and aids:

- language barriers, cultural difference, not appropriate for all,
- family conflict, their lack of understanding or misconceptions,
- available time to discuss with patient and family,
- patient not emotionally ready for discussions,
- patient decision aid was too condition specific; too rigid,
- patients/families not accepting nursing support on this (not their role).

Many nurses commented that they had limited opportunities to use the patient decision aid for CPR, but did identify that they used patient decision support for other issues. Specifically, some nurses commented on identifying decisional conflict and validating patients' values. A few nurses stated that CPR status should be determined on admission to hospital and be completed routinely for all patients. There were varied views regarding evaluating CPR status on admission versus at a time of health crisis. Some said it should be addressed for every patient, despite health status, and some indicated that only when death may be imminent it should be discussed. Some nurses stated that they did not see this as a part of their role or something that they wish to partake in. Others thought that this was completely within the nursing realm and were eager to support patients with making an informed CPR choice. Most nurses agreed with the components of a shared decision making model.

### 6.4. Findings versus Literature

After reviewing the data collected it was evident that most nurses were willing to use the patient decision aid because they see it as helping the patient make informed, value-based decisions. The findings were consistent with the literature [13, 23, 38]. When nurses feel they have the knowledge and skill to provide decision support they do so because they believe that they are helping the patient toward a better realization of their condition. The education session and patient decision aid intervention did not seem to significantly influence questionnaire scores, but it was observed with qualitative observations that the nurses felt more knowledgeable and confident with providing patient decision support. 

### 6.5. Barriers and Facilitators

Specific barriers to providing patient decision support were identified as cultural or language influences, time constraints, rigid application, patient's emotional adversity, and physician preference for this role. These mirror what has been found in the literature [13, 23, 36]. Specific facilitators identified included communication enhancement, clear and understandable knowledge base, and the ability to include family in the decision making process. There is a limited amount of literature that describes patient decision support facilitators and even less focus on family involvement [23]. This could be a spotlight for future quality improvement interventions.

### 6.6. Limitations

 Data were collected from self-report and observations, not from a validated tool; thus obvious sources of bias were present. The information collected was helpful in this specific clinical setting but cannot be generalized to others. Time was also a limiting factor. There were only six weeks where the patient decision aid was implemented and the opportunity for its use did not come readily. Most nurses welcomed this intervention but some were obviously stressed at the fact that they were approached to participate as evidenced by their body language and facial expressions. This intervention was not the only quality improvement project being initiated. This project may have been better received during a less demanding time for nurse involvement. 

### 6.7. Implications for Practice

This project identified that CPR status specifically can be appraised by a nurse to be a difficult topic, too patient specific to use a patient decision aid, or confident that this would be used as a guide to improve patients' knowledge of options and the provision of support. CPR status is value-sensitive topic, but it is not beyond what normally would be encountered by a practicing nurse. Addressing unclear values, information needs, and resources effectively will reduce nursing contributing factors to clouding difficult decisions [5, 11]. Patient decision aids are designed to be evidenced based and patient focused and are intended to be used as a guide [12]. Daily nursing practice should reflect a therapeutic relationship between the patient and nurse toward helping the patient make informed and supported decisions through the provision of patient decision support. Nursing perceptions need to focus on positive implications.

## 7. Conclusion

Based on this quality improvement project, a practice change towards supporting patients to be more educated and involved in their decision making is a priority. Since nurses work in close proximity with the patient and their families and spend much time involved in their care, they are the most appropriate professionals to discuss CPR preferences using a shared-decision making model [23, 36, 44]. Further education on when to implement a patient decision aid for values sensitive topics would be appropriate. Strategically educating certain nurses on patient decision support may create an effective role for its implementation. Over time challenging traditional health care roles will allow nurses to support the patient effectively through the provision of patient decision support. 

Challenging the barriers to implementing patient decision support and enhancing the facilitating factors will eventually disclose the benefits of its use. This project identified some of those factors within the Acute Monitor Area at The Ottawa Hospital. Dedication and commitment to supporting patient decision support and the cardiopulmonary resuscitation decision aid will help to support patients facing these difficult situations. 

## Figures and Tables

**Table 1:** Before CPR patient decision aid questionnaire results.

Statements	Strongly disagree *n* = 16 (%)	Disagree *n* = 16 (%)	Neutral *n* = 16 (%)	Agree *n* = 16 (%)	Strongly agree *n* = 16 (%)
Most patients prefer to make decisions on their own	—	6 (29%)	1 (5%)	12 (57%)	2 (9%)
Most patients prefer to make decisions withothers	—	—	—	20 (95%)	1 (5%)
Most patients prefer to make decisions after considering their health care team's opinions	—	—	4 (19%)	15 (71%)	2 (9%)
Patient decision support will increase patient involvement in making health decisions	—	—	3 (14%)	15 (71%)	3 (14%)
Nurses generally feel confident about providing patient decision support	1 (5%)	4 (19%)	8 (38%)	8 (38%)	—
Nurses understand patient decision support concepts	—	—	4 (19%)	16 (76%)	1 (5%)
Nurses need to increase their knowledge of decision support	—	1 (5%)	1 (5%)	14 (67%)	5 (24%)
Nurses need to enhance their ability to provide patient decision support	—	—	2 (9%)	16 (76%)	3 (14%)
There should be more education on patient decision support/aids	—	—	—	16 (76%)	5 (24%)
I feel more education on patient decision support/aid would benefit the patient	—	—	—	16 (76%)	5 (24%)

**Table 2:** After CPR patient decision aid questionnaire results.

Statements	Strongly disagree *n* = 16 (%)	Disagree *n* = 16 (%)	Neutral *n* = 16 (%)	Agree *n* = 16 (%)	Strongly agree *n* = 16 (%)
Most patients prefer to make decisions on their own	—	4 (25%)	2 (13%)	8 (50%)	2 (13%)
Most patients prefer to make decisions withothers	—	—	2 (13%)	7 (44%)	7 (44%)
Most patients prefer to make decisions after considering their health care team's opinions	—	—	1 (6%)	13 (81%)	2 (13%)
Patient decision support will increase patient involvement in making health decisions	—	—	2 (13%)	8 (50%)	6 (38%)
Nurses validate patient's values when providing patient decision support	—	—	3 (19%)	12 (75%)	1 (6%)
Patients should be referred to a specialized nurse educated in decision support	—	—	5 (31%)	10 (63%)	1 (6%)
Nurses generally feel confident about providing patient decision support	—	2 (13%)	8 (50%)	6 (38%)	—
The patient decision aid is a good resource (e.g., easy to understand, or nonbiased)	—	—	2 (13%)	13 (81%)	1 (6%)
The decision aid was easily applied to the clinical setting	—	1 (6%)	7 (44%)	8 (50%)	—
There was clear direction in providing patient decision support to patients with the CPR decision aid	—	1 (6%)	6 (38%)	9 (56%)	—
Nurses prefer to have a clear step-by-step approach when supporting patients on deciding CPR status	—	2 (13%)	3 (19%)	10 (63%)	1 (6%)
The decision aid made it easier for nurses to identify patients having difficulty in making a CPR choice	—	—	4 (25%)	12 (75%)	—
Overall, I feel that patient decision support/aids for CPR status is useful	—	—	3 (19%)	10 (63%)	3 (19%)
